# Development and Validation of a Comprehensive Questionnaire to Assess Interpersonal Discord (Bullying, Harassment, and Discrimination) at the Workplace in a Healthcare Setting

**DOI:** 10.7759/cureus.18467

**Published:** 2021-10-03

**Authors:** Amandeep Singh, Piyush Ranjan, Tanveer Kaur, Siddharth Sarkar, Ashish D Upadhyay, Upendra Baitha, Prayas Sethi, Ranveer S Jadon, Pankaj Jorwal

**Affiliations:** 1 Medicine, All India Institute of Medical Sciences, New Delhi, IND; 2 Addiction, All India Institute of Medical Sciences, New Delhi, IND; 3 Statistics, All India Institute of Medical Sciences, New Delhi, IND; 4 Internal Medicine, All India Institute of Medical Sciences, New Delhi, IND

**Keywords:** bullying, health personnel, tool, workplace discord, workplace violence

## Abstract

Objective

This study was conducted to develop and validate a comprehensive questionnaire to assess bullying, discrimination, and harassment in healthcare settings.

Methodology

A mixed-method study design was used to develop and validate the questionnaire. In phase I, qualitative approaches were used for the development, which included literature search, focus group discussions (FGDs), following which the construct was developed. In phase II, face validity and construct validity were established using quantitative approaches.

Results

The final questionnaire consists of 25 items divided into five sections addressing the burden, impact, reasons for underreporting, risk factors, and mitigation strategies. The questionnaire has very good consistency with a Cronbach’s alpha score of 0.86.

Conclusion

This is a comprehensive tool with appropriate psychometric properties with potential use for evaluating the problem of interpersonal discord in the form of bullying, harassment, and discrimination in a healthcare setting.

## Introduction

The phenomenon of workplace discord has a daunting effect on all sectors [1]. Although it has not spared any work setting, the healthcare sector appears to be one of the worst affected [2]. Discord is defined as any form of interpersonal disharmony, and it mostly occurs in the form of bullying, harassment, and discrimination at the workplace [3]. Studies suggest that almost three in four healthcare personnel have experienced some form of discord in their career [4,5]. Such incidents disrupt the occupational environment by creating disharmony and have a detrimental effect on the physical, emotional, social, and psychological well-being of those who are at the receiving end [6]. It causes a decline in job performance, burnout, absenteeism, etc. Similarly, its impact is also reflected in the organizational environment, in the form of a hostile or toxic environment, which directly leads to decreased patient safety and quality of care. It obstructs communication and disrupts effective teamwork, which increases medical errors by affecting the quality of healthcare organizations [7].

The tolerance, reporting, and mitigation strategies for workplace discord vary in different countries and cultures based on the cultural and psychological conditioning of its healthcare personnel [8]. Validated tools are available to assess bullying, harassment, and discrimination, but they lack comprehensiveness due to their individual-oriented or incomplete nature [9,10,11]. Besides, they lack appropriate psychometric properties as well [12]. Hence there is a need to develop and validate a single, concise, and comprehensive evaluation tool to assess the genesis and spectrum of the problem and to devise strategies to combat it from individual to policy levels.

## Materials and methods

Study design and ethical consideration

**Figure 1:**
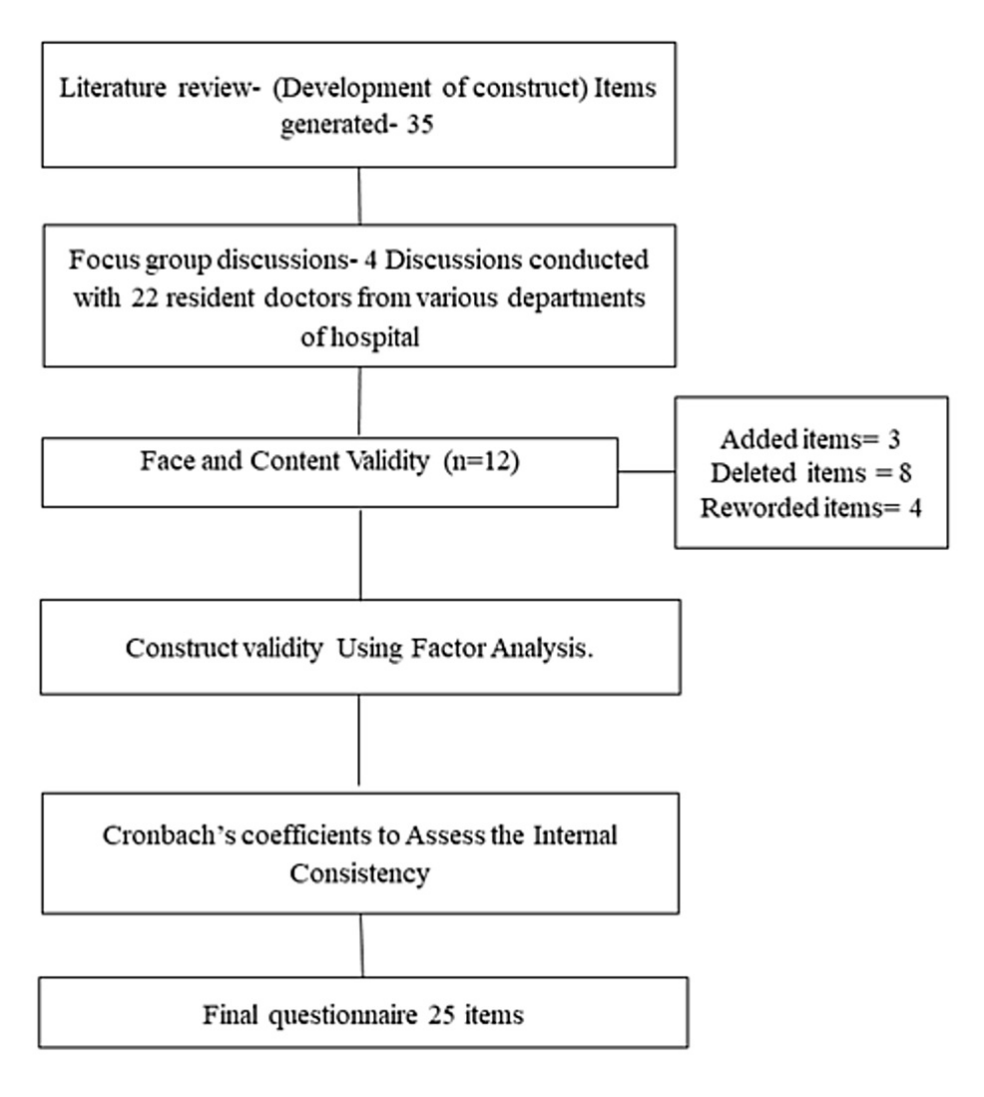
Flowchart for methodology

The development and validation of the questionnaire were performed using a mixed-method design with standardized techniques (Figure 1) [13,14,15]. The study was approved by the Institute Ethics Committee of the All India Institute of Medical Sciences, New Delhi (IEC-844/06.12.2019, RP-46/2020). The informed written consent was taken from all the participants and confidentiality and anonymity were assured.

Phase 1: Development of the questionnaire

In this phase, a systematic methodology was employed by the incorporation of the following key steps: literature review, focus groups discussions (FGDs), expert evaluation, and pre-testing.

The first step included a comprehensive and exhaustive literature search, which was done using a search string (aggression OR violence OR bullying OR harassment OR abuse OR discrimination) AND (surgeon OR resident OR intern OR physician OR doctor OR “general practitioner” OR “healthcare” OR clinicians) AND (workplace) AND (“risk factor” OR predictor OR determinants) AND (prevent* OR strategy* OR intervention) on PubMed and Wiley. The initial search resulted in 714 articles, from which 18 relevant articles were selected. A total of 32 items were generated in this step.

In the following step, four FGDs were conducted with resident doctors, nurses, and faculty members (n=22), each having a minimum of five and a maximum of seven participants from various departments of the hospital. Seven items were generated in this step. The duplicate items were removed, and the final pool of questionnaires with 30 items was categorized into the following five domains: forms of discord, the impact of discord, reporting of discord, mitigation strategies for discord, and risk factors of discord. Emphasis was laid on keeping the language of the questionnaire simple, clear, and unambiguous.

Phase 2: Validation of the questionnaire

For expert validation, qualitative and quantitative approaches were used. A panel of 12 experts was invited to evaluate the qualitative validity of the questionnaire and comment on the correctness and quality of the items. Items were changed or reworded as per the inputs. For quantitative content validity, the content validity ratio (CVR) and content validity index (CVI) were derived. The experts evaluated the items based on need, clarity, and relevance. The usefulness of items was graded on a 3-point scale, with scores ranging from 1 (not required), 0 (helpful but not essential), and +1. (essential). The CVR formula is CVR=(Ne-N/2)/(N/2), where Ne is the number of participants who marked an item as essential, and N is the total number of participants [16]. The appropriate CVR values were calculated using the Lawshe scores. Each item's relevance and clarity were also determined using a 4-point Likert scale: (1) not relevant/clear, (2) marginally relevant/clear and need revision, (3) relevant/clear and requires minimal revision, and (4) very relevant/clear. The proportion of experts who rated an item as relevant/clear/simple determined its CVI (ratings of 3 or 4) [17]. Items with a CVI value of less than 0.7 were removed, and those with a score between 0.7 and 0.79 were changed based on expert advice [17]. After this, the questionnaire with 25 items was pre-tested on 20 participants, and further changes were implemented as per their suggestions. The questionnaire was modified by adding (three items), deleting (eight items), and rewording (four items) items based on their suggestions.

Following this, a web-based questionnaire was distributed in August 2021, through a shared web link. The participants were informed about the purpose of the study through a 'Participant Information Sheet', and their responses were recorded by ensuring anonymity and prior consent. A total of 130 resident doctors, faculty members, and other healthcare workers from various departments of the hospital completed the questionnaires. The data was collected through a convenience sampling method, and the principle of maximum diversity was ensured.

Statistical analysis

The analysis of qualitative demographic variables such as gender, education status, and occupation was done using descriptive statistics. For the quantitative variables, mean, median, standard deviation, quartile, and range were calculated. The internal consistency (i.e., the range at which the items on the instrument measure the same thing) of the questionnaire was assessed with Cronbach's alpha. Good internal consistency was indicated by a Cronbach alpha score of 0.7 or higher. Exploratory factor analysis was done to evaluate the subdomain structure. The aim of this technique was the estimation of factors and to reduce the dimensionality of a large number of variables to a fewer number of factors. The sample adequacy was measured by the Kaiser-Meyer-Olkin (KMO) test, and values of more than 0.5 showed that the data were suitable for factor analysis. Bartlett's test of sphericity is a statistical test for the overall significance of all correlations within a correlation matrix. Eigenvalues represent the variance in the variables that is accounted for by a specific factor.

## Results

The questionnaire comprises 25 items, which are divided into the following five domains: forms of discord (four items), the impact of discord (five items), reporting of discord (eight items), mitigation strategies for discord (four items), and risk-factors of discord (four items). In the first domain, the questions are specific to the spectrum and prevalence of workplace-based discord; they are designed with caution to capture aspects of bullying, harassment, and discrimination based on race, community, religion, and culture. In the second domain, the questions are focused on understanding the impact of such episodes on personal and social life. Even though the incidences of workplace discord are very high in all parts of the world, many significant findings suggest that the reporting of such incidents is very low [18]. Hence the third domain deals with the reasons related to underreporting of such incidents from individual to organizational levels. In the fourth domain, questions related to mitigation strategies are also added, which focus on complaint redressal, legal rights, and responsibilities, improving management facilities, and strong legislative measures, and in the fifth domain, questions related to risk factors specific to the scenario of the country to address the problem holistically are dealt with.

Sociodemographic profile of the participants

The sociodemographic characteristics of 130 participants included in the validation phase are presented in Table 1. The mean age of the participants was 29.31 ± 4.64 years. The entire sample worked in a government setting and had a higher proportion of males (63%) as compared to females (36%). The majority of them hailed from metropolitan cities (92%). There was a fair representation from all departments of the healthcare setting.

**Table 1:** Sociodemographic profile of the participants (n=130) MBBS: Bachelor of Medicine, Bachelor of Surgery; SD: standard deviation

Characteristics		N	%
Age in years, mean ± SD	29.31 ± 4.64		
Gender	Male	82	63.08
	Female	47	36.15
	Prefer not to say	01	00.77
	Others	00	0.00
Designation	MBBS/BSc	21	16.15
	MD/MSc	90	69.23
	MD/Ph.D	19	14.62
Workplace setting	Government hospital	130	100
Area	Metropolitan	120	92.31
	Urban	9	06.92
	Rural	1	00.77
Number of years of experience after completion of MBBS/BSc (internship counted as the first year of experience), mean ± SD	5.83 ± 4.53		
Department of residency/specialization/working	Emergency	22	16.92
	Medicine	42	32.30
	Surgery and allied	08	06.15
	OBS	28	21.53
	Pediatrics	01	00.77
	Trauma	11	08.46
	Anesthesia and critical care	03	02.31
	Others	15	11.54
Marital status	Married	32	24.62
	Unmarried	97	74.62
	Others	01	00.77

Validity of the questionnaire

To screen out the intercorrelation and singularity between items, the intercorrelation matrix was used. In the screening process, researchers found only three items (6, 7, and 8) showing intercorrelation above 0.70. But these were not excluded from the final draft of the questionnaire as these were found to be important items regarding the impact of bullying at the workplace. Cronbach's alpha is considered one of the most effective reliability tests. It helps in finding out the internal consistency of any questionnaire. In our study, it was found to be good, i.e., α=0.86. Principal component factor analysis along with Varimax rotation was run to establish the sampling adequacy and factorial validity of the questionnaire. After running the principal component factor analysis, the factorial validity was found to be 73.66, which is satisfactory. Additionally, the KMO value (0.766) and Bartlett's test of sphericity (p<0.01) determined the adequacy of the sample. The intercorrelation matrix is presented in Table 2.

**Table 2:** Intercorrelation matrix

	Q1	Q2	Q3	Q4	Q5	Q6	Q7	Q8	Q9	Q10	Q11	Q12	Q13	Q14	Q15	Q16	Q17	Q18	Q19	Q20	Q21	Q22	Q23	Q24	Q25
Q1	1																								
Q2	0.437	1																							
Q3	0.348	0.613	1																						
Q4	0.267	0.551	0.352	1																					
Q5	0.387	0.23	0.167	0.311	1																				
Q6	0.317	0.25	0.216	0.209	0.469	1																			
Q7	0.433	0.199	0.171	0.139	0.513	0.734	1																		
Q8	0.363	0.301	0.222	0.193	0.515	0.754	0.803	1																	
Q9	0.45	0.321	0.366	0.309	0.642	0.687	0.605	0.603	1																
Q10	0.341	0.291	0.223	0.345	0.244	0.105	0.139	0.119	0.302	1															
Q11	0.129	0.195	0.075	0.127	0.039	0.117	0.089	0.108	0.209	0.112	1														
Q12	0.309	0.126	0.202	0.293	0.37	0.176	0.217	0.236	0.265	0.345	0.239	1													
Q13	0.297	0.2	0.265	0.325	0.365	0.215	0.298	0.319	0.291	0.305	0.176	0.839	1												
Q14	0.042	-0.001	0.023	-0.051	0.026	0.159	0.156	0.15	0.133	0.056	0.303	0.329	0.354	1											
Q15	0.277	0.246	0.259	0.229	0.3	0.23	0.23	0.256	0.236	0.151	0.163	0.546	0.609	0.348	1										
Q16	0.147	0.159	0.086	0.131	0.221	0.315	0.282	0.307	0.168	0.058	0.265	0.549	0.541	0.201	0.663	1									
Q17	0.268	0.191	0.143	0.192	0.264	0.262	0.274	0.262	0.338	0.183	0.331	0.579	0.507	0.304	0.531	0.548	1								
Q18	-0.137	-0.102	-0.099	-0.15	-0.139	-0.077	-0.074	-0.157	-0.052	-0.174	-0.137	-0.317	-0.216	-0.105	-0.254	-0.123	-0.317	1							
Q19	0.084	0.141	0.118	-0.003	0.021	-0.017	0.03	0.094	0.089	0.083	0.247	0.164	0.162	0.036	0.179	0.068	0.284	-0.573	1						
Q20	-0.085	0.082	0.119	0.003	0.079	0.024	0.007	0.075	0.053	0.056	0.077	0.129	0.127	0.087	0.187	0.053	0.16	-0.536	0.605	1					
Q21	0.011	0.113	0.01	0.163	0.181	0.084	0.003	0.131	0.041	-0.093	0.236	0.253	0.137	0.17	0.221	0.203	0.229	-0.502	0.292	0.499	1				
Q22	0.082	0.138	0.076	0.029	0.061	0.022	0.032	0.121	0.071	0.063	0.234	0.238	0.309	0.185	0.307	0.275	0.338	-0.172	0.359	0.184	0.135	1			
Q23	0.049	0.028	0.036	-0.138	-0.083	0.028	0.096	0.145	-0.012	-0.008	0.183	0.224	0.256	0.386	0.211	0.176	0.12	-0.177	0.255	0.213	0.165	0.493	1		
Q24	0.088	0.264	0.192	0.146	0.043	0.035	0.071	0.223	0.039	0.147	0.265	0.288	0.269	0.287	0.258	0.272	0.108	-0.153	0.113	0.216	0.28	0.348	0.425	1	
Q25	0.029	0.135	0.042	0.113	0.189	0.023	-0.022	0.067	0.103	0.088	0.167	0.263	0.259	0.306	0.277	0.16	0.213	-0.184	0.192	0.289	0.503	0.364	0.342	0.531	1

## Discussion

The healthcare system and its workers are the cornerstones of a country as they contribute significantly to its development and prosperity. Their mental, physical, emotional, and social wellbeing are of utmost importance as it directly affects their work efficiency, motivation, turnover intention, and quality of life [19]. The rising incidents of discord at the workplace have motivated various researchers to develop tools to assess the magnitude of the problem and to devise strategies to address it systematically [20,21,22].

Globally, various studies have been conducted to assess the problem of bullying, harassment, and discrimination in the healthcare sector. But most of them have assessed these problems separately and used semi-structured interview techniques, which makes it difficult to perform a comparative analysis between different studies [12]. Additionally, they either have low psychometric properties or lack comprehensiveness as a single scale to capture various domains. While we constructed a questionnaire on various aspects that affect interpersonal discord in healthcare settings, we extensively reviewed the existing data and based on that, various domains of bullying, harassment, and discrimination were identified. The questionnaire is free to use and is presented in Table 3.

**Table 3:** Questionnaire for workplace discord (bullying, harassment, and discrimination) scale in healthcare settings

Name:		
Age (in years):	Gender:	
Highest degree:	Workplace setting:	
Area of workplace:	Marital status:	
Department of residency/specialization/working:		
Number of years of experience after completion of MBBS/BSc (count Internship as first year of experience): _________________		
Section A: Forms of discord		
Item numbers 1-5 intend to assess the frequency of various forms of discord (bullying, harassment, and discrimination) experienced by healthcare workers in healthcare settings. Mark the most appropriate option		
A1: How often do you get bullied (e.g., mockery, insult, etc. by your colleagues and/or seniors) at your workplace?		
Nearly daily		
About once a week		
About once a month		
About once every six months		
About once a year or less		
A2: How often do you experience harassment/discrimination based on race/community/caste/religion at your workplace?		
About once in a month or more		
About once every six months		
About once a year		
Less than once a year		
Never		
A3: How often do you experience harassment/discrimination based on your state of origin at your workplace?		
About once in a month or more		
About once every six months		
About once a year		
Less than once a year		
Never		
A4: “As per the guidelines, sexual harassment includes any unwelcome sexually determined behavior (whether directly or by implication) as: a) physical contact and advances; b) a demand or request for sexual favors; c) sexually colored remarks; d) showing pornography; e) any other unwelcome physical, verbal, or non-verbal conduct of sexual nature.” How often does an incident of sexual harassment at your workplace come to your knowledge?		
About once in a month or more		
About once every six months		
About once a year		
Less than once a year		
Never		
Section B: Impact of incidences of discord		
Following are the statements/questions regarding the effect of discord (like bullying, harassment, and discrimination) at your workplace on the different aspects of life. Mark the most appropriate response among the options given below with each statement:		
B1: On the basis of the episodes of discords (like bullying, harassment, and discrimination) at my workplace, I have developed the following feelings: ______		
Not affected		
Mildly affected		
Moderately affected		
Severely affected		
Very severely affected		
B2: Personal wellbeing and self-care include activities such as sleep schedule, eating pattern, fitness, grooming, dressing, etc. How much have the episodes of discords (i.e., bullying, harassment, and discrimination) at your workplace affected your personal wellbeing and self-care?		
Not affected		
Mildly affected		
Moderately affected		
Severely affected		
Very severely affected		
B3: “Family life is defined as the routine interactions and activities that a family have together especially with the members who live together, such as parents, spouse, children.” How much has your family been affected due to the episodes of discords (i.e., bullying, harassment, and discrimination) at your workplace?		
Not affected		
Mildly affected		
Moderately affected		
Severely affected		
Very severely affected		
B4: “Social life is defined as the part of a person's time spent doing enjoyable things with others like friends, colleagues, or people living in the society other than close family members.” How much has your social life been affected due to the episodes of discords (i.e., bullying, harassment, discrimination) at your workplace?		
Not affected		
Mildly affected		
Moderately affected		
Severely affected		
Very severely affected		
B5: How much have the episodes of discords (i.e., bullying, harassment, discrimination) at your workplace affected your mental and psychological wellbeing (increased aggressiveness, irritability, low self-esteem, etc.)?		
Not affected		
Mildly affected		
Moderately affected		
Severely affected		
Very severely affected		
Section C: Reporting of incidence		
This domain assesses how comfortable or confident workers are about reporting the incidences of discord to the higher authorities. C1: Are you comfortable in reporting the incidences of discords (bullying, harassment, and discrimination) at your workplace to the authorities?		
Significantly		
Somewhat significantly		
Insignificantly		
The statements given below are some of the reasons why the incidences of violence are underreported to the authorities. To what extent do these following reasons lead to underreporting? Select the most appropriate choice in your opinion. C2: Feeling ashamed of reporting the incidences of discords (bullying, harassment, and discrimination) at the workplace		
Significantly		
Somewhat significantly		
Insignificantly		
C3: A belief that no action will be taken against the perpetrator		
Significantly		
Somewhat significantly		
Insignificantly		
C4: Lack of organizational support		
Significantly		
Somewhat significantly		
Insignificantly		
C5: Believe that violence is a part of our job		
Significantly		
Somewhat significantly		
Insignificantly		
C6: Lack of provision to report such incidences		
Significantly		
Somewhat significantly		
Insignificantly		
C7: The process of reporting is/was time-consuming		
Significantly		
Somewhat significantly		
Insignificantly		
C8: Fear that the appraisal or promotion avenues will be affected		
Significantly		
Somewhat significantly		
Insignificantly		
Section D: Mitigation strategies		
This domain focuses on the strategies that can be useful in preventing episodes of discord at the workplace. The statements given below are some of the strategies that can check the episodes of discord (bullying, harassment, and discrimination) at the workplace. To what extent do you think that the respective strategy can be useful in preventing such episodes?		
D1: Regular training of healthcare workers regarding their legal rights and responsibilities		
Very useful		
Somewhat useful		
Not useful		
D2: Improved management policies (e.g., 360-degree evaluation/bi-directional appraisal, where the boss and the subordinates can provide feedback for each other)		
Very useful		
Somewhat useful		
Not useful		
D3: Effective complaint redressal system		
Very useful		
Somewhat useful		
Not useful		
D4: Strong legislative measures like provision of significant punishment for offenders		
Very useful		
Somewhat useful		
Not useful		
Section E: Risk factors related to incidents of workplace discord		
This section intends to assess the factors that might be an important reason in the genesis/continuation of the episodes of discord (bullying, harassment, and discrimination) in healthcare settings		
E1: Institutional attributes: This includes dominance due to hierarchy between the boss and the employees. Tolerance towards discord has been an accepted norm in the work environment over the years. How much (in your opinion) do the Institutional attributes contribute to the genesis/continuation of such episodes at the workplace?		
Very important		
Somewhat important		
Not important		
E2: Interpersonal attributes: Misbehaving with someone or tolerating an episode of discord may be a personal attribute. There is always some difference in the inherent nature and behavior of individuals to initiate or tolerate things like bullying, harassment, and discrimination. How much (in your opinion) do the Interpersonal attributes contribute to the genesis/continuation of such episodes at the workplace?		
Very important		
Somewhat important		
Not important		
E3: Social attributes: Social exclusion/marginalization of individuals due to discrimination on the basis of race/ethnicity/community/social status, etc. How much (in your opinion) do the social attributes contribute to the genesis/continuation of such episodes at the workplace?		
Very important		
Somewhat important		
Not important		
E4: Legislative attributes: It may include factors like lack of stringent laws and policies, inadequate redressal system, etc. How much (in your opinion) do the legislative attributes contribute to the genesis/continuation of such episodes at the workplace?		
Very important		
Somewhat important		
Not important		

A heterogenous Likert scale was used to scale the responses based on the intensity or severity of the problem in each domain. Section A comprises items related to forms of discord like bullying, harassment, and discrimination based on race/caste/community, as well as the state of origin. Along with this, the issue of sexual harassment has also been given due importance, as these forms of non-physical violence are more common and mostly overlooked [23]. Since such episodes affect all aspects of an individual's life, Section B has focused on its impact on personal, familial, social, and psychological wellbeing as well. Despite a high prevalence, the discord cases are often underreported and normalized [24]; therefore, in Section C, the prime focus was on assessing their reasons for not reporting such events, which included questions related to their psychological conditioning and organizational dynamics redressal system. In Section D, the focus was placed on solving the problem by incorporating changes in the training, legislation, redressal system, etc. Lastly, Section E intends to highlight the various risk factors associated with discord, which includes institutional, interpersonal, social, and legislative attributes. This questionnaire represents a way forward to tackle the problem of bullying, harassment, and discrimination, as it is likely to provide baseline parameters to work upon. It will help identify not just the frequency or severity of the problem but its risk factors, impact, and mitigation strategies that will help in implementing changes from individual to policy levels. This will also aid in conducting large cross-sectional studies for analyzing the contrast in workplace discord among various countries or within a country across various settings.

Strengths and limitations

The questionnaire we devised is easy to administer and addresses the issues of discord in a simple and clear manner. It is scientific and psychometrically evaluated and, to our belief, will be useful in assessing a wide range of issues from the spectrum and prevalence to the mitigation strategies for workplace discord from individual to policy levels. The major limitation is the semi-quantitative nature of the questionnaire and the fact that it lacks the assessment of predictive validity.

## Conclusions

Despite increased awareness and having various laws and measures in place, discord in the healthcare setting persists, and there are significant factors that pose as barriers to reporting and eradicating the problem. Gaining awareness and knowledge about the most prevalent behaviors will aid in the development of interventions targeted at the most problematic negative behaviors. The questionnaire we developed is a reliable and valid tool to assess bullying, harassment, and discrimination in the healthcare setting. It is clear, concise, and easy to administer. It has the potential to dismantle the normalization of discord at the workplace and will contribute greatly to the existing literature on the topic.
